# ZEB1 in Pancreatic Cancer

**DOI:** 10.3390/cancers2031617

**Published:** 2010-08-18

**Authors:** Ulrich Wellner, Thomas Brabletz, Tobias Keck

**Affiliations:** Department of General and Visceral Surgery, University of Freiburg, Hugstetter Straße 55, 79106 Freiburg, Germany; E-Mails: ulrich.wellner@uniklinik-freiburg.de (U.W.); thomas.brabletz@uniklinik-freiburg.de (T.B.)

**Keywords:** pancreatic cancer, ZEB1, EMT, MCSC

## Abstract

Pancreatic cancer is one of the most malignant human neoplasias. On the molecular level, epithelial-mesenchymal transition (EMT) has been demonstrated to contribute to the malignant phenotype of pancreatic cancer cells. ZEB1 is a transcriptional repressor that has been identified as an inducer of EMT. A negative feedback loop between ZEB1 and microRNA-200c has been shown to regulate this EMT induction in various models. With respect to pancreatic cancer, primary effects of EMT comprise increased local and distant tumor cell dissemination. Another recently described feature of the EMT is the acquisition of cancer stem cell traits. For pancreatic cancer cells, antagonism between ZEB1 and stemness-inhibiting micro-RNAs has been demonstrated to contribute to this process, providing experimental support for the migrating cancer stem cell (MCSC) hypothesis. ZEB1 has also been shown to be associated with drug resistance of pancreatic cancer cells. This article reviews the biological functions of ZEB1 with a focus on pancreatic cancer.

## 1. Introduction

Epithelial-mesenchymal transition (EMT) is a process that was first discovered in development. During EMT, epithelial cells lose their apicobasal polarity and tight cell-cell contacts and acquire a fibroblast-like migratory phenotype. This is accompanied by a loss of epithelial markers like E-Cadherin and expression of mesenchymal markers like Vimentin [1,2,3,4,5,6].

Apart from its role in development, EMT has recently been found to contribute to migration and invasion of carcinoma cells and the formation of metastasis. Furthermore, there is mounting evidence that EMT can induce cancer stem cell properties including tumorigenicity and drug resistance.

The ZEB proteins (ZEB1 and ZEB2) are closely related transcription factors. Their best described biological function is the induction of EMT in epithelial cells. Here we review the biological functions of ZEB1 and its role in cancer, especially pancreatic cancer.

## 2. Structure-Function Relations of the ZEB1 Molecule

**Figure 1:**
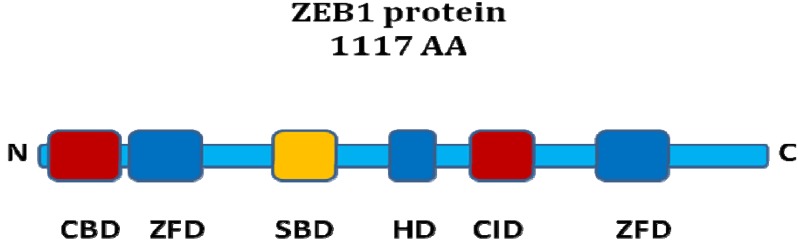
ZEB1 protein structure. Schematic representation of functional domains of the ZEB1 protein. ZFD: zinc finger domain, HD: homeodomain, SBD: Smad binding domain, CID: CtBP interaction domain, CBD: Coactivator binding domain. The ZFDs bind to DNA. The corepressor CtBP binds to the CID whereas the coactivators p300 and P/CAF interact with the CBD. R-Smads bind *via* the SBD.

There are two homologous ZEB proteins in vertebrates (ZEB1 and ZEB2) but only one ortholog in *Drosophila melanogaster* (Zfh-1) and *Caenorhabditis elegans* (Zag-1). A simplified schematic of the ZEB1 protein is shown in Figure 1.

The ZEB proteins contain two C2H2-type zinc finger domains (ZFD) and a central POU-like homeodomain (HD). There is around 90% sequence homology between ZEB1 and ZEB2 in the ZFD, but much less in the other domains. DNA-binding occurs *via* the ZFDs, which recognize specific sequences in the promoter region of ZEB target genes [7]. ZEB1 binds to Z-boxes and E-boxes. Both ZEB proteins undergo posttranscriptional SUMOylation, affecting the repressive activity [8].

The ZEB proteins can bind to activated R-Smads *via* a Smad-binding domain (SBD). An antagonism regarding the TGF-beta pathway has been suggested: while ZEB1 acts synergistically with R-Smads to activate TGF-beta target gene transcription and growth arrest, ZEB2 seems to have the opposite function. This antagonism was postulated to be due to differential recruitment of coactivators (p300 and P/CAF) by ZEB1 *versus* the corepressor CtBP1 by ZEB2 [9].

The interaction of ZEB proteins with CtBP occurs via a C-terminal CtBP interaction domain (CID) containing PXDLS-motifs. Both ZEB1 and ZEB2 have been found to bind CtBP1 [10,11], but ZEB1 binds CtBP1 less efficiently than ZEB2. Direct interaction of ZEB1 with CtBP2 and HDAC1 has also been shown to contribute to transcriptional repression [12,13,14]. ZEB1 and ZEB2 are the DNA-binding members of the CtBP core complex for transcriptional repression of E-Cadherin [15], and this complex also contains the corepressors CtBP1 and CtBP2, the histone-deactylases HDAC1 and HDAC2, as well as methyltransferases. As ZEB1/2-mediated repression of E-Cadherin has also been found to be possible without CtBP1, the recent discovery of BRG1 as an alternative corepressor for ZEB1 is noteworthy [16].

## 3. ZEB1 in Development

During mouse embryonic development, ZEB1 is expressed in various tissues like the notochord, neural crest derivatives, restricted sites of the central nervous system (CNS), somites and limb. [17,18]. Homozygous ZEB1^−/−^ knockout mice die perinatally of respiratory failure and show various skeletal defects as well as severe T cell deficiency of the thymus [18]. This is in line with the observation that ZEB1 controls collagen expression in chondrocytes and osteoblasts [19,20] as well as CD4 and IL2 expression in T-lymphocytes [12,21]. In contrast to ZEB2^−/−^ knockout mice, there are no specific defects in the central nervous system, suggesting a compensatory role of ZEB2, which is widely expressed in the developing mouse CNS. Compound ZEB1^−/−^ ZEB2^−/−^ mice, display a severe failure of neural tube closure highlighting the importance of ZEB-induced EMT [22].

In the *Xenopus laevis* embryo, XdEF1 (ZEB1 ortholog) is expressed during neurulation in the paraxial mesoderm. Coexpression of XdEF1 and XSIP1 (ZEB2 ortholog) is found in the migratory neural crest, neural tube and retinal cells.

## 4. ZEB1 in Cancer

### 4.1. ZEB1 Targets in Cancer

After characterization of its functions in development, a role for ZEB1 in EMT of cancer cells, invasion and metastasis became a focus of research. Interestingly, the anti-anoikis and anti-metastatic effect of adenovirus E1a led to the discovery of the direct transcriptional repression of E-Cadherin by ZEB1 in human cancer cells [23,24]. Another early study found ZEB1 to be upregulated in the context of Snail-induced EMT in human carcinoma cells [25]. ZEB1 expression remained elevated for more than 20 days after withdrawal of Snail. Therefore, the biological role of ZEB1 might be to stabilize the mesenchymal phenotype after transient expression of other EMT-inducers like Snail. In panels of cancer cell lines including the NCI-60, ZEB1 expression showed a much stronger negative correlation with E-Cadherin expression than ZEB2, Slug, Snail or Twist [24,25,26,27].

The first comprehensive study of potential ZEB1 targets by whole genomic cDNA array analysis revealed a master regulator function of ZEB1 in epithelial cell plasticity [28]. According to its predominant repressor function, about 200 genes were found to be downregulated on mRNA level by ZEB1, compared to only 30 upregulated genes. The list of repressed targets comprises epithelial cell polarity genes, classical cadherins, desmosome proteins, tight and gap junction proteins, apical and vesicle transport proteins as well as epithelial cell surface receptors.

A negative feedback loop between ZEB1 and microRNA-200 family has recently been described in various carcinoma models [29,30,31,32,33,34]. This feedback loop could constitute a molecular basis for stabilization of either the epithelial or the mesenchymal state after induction of an EMT/MET process. ZEB1 can repress transcription of the miR-200c-141 and the miR-200b-200a-429 polycistronic pri-miR transcripts by direct binding to the promoter regions [31,32]. *Vice versa*, ZEB1 is a target of the microRNA-200 family. Its seems that of this family, miR-200c is has the strongest anti-ZEB1 and anti-EMT effect [30]. 

### 4.2. Biologic Effects Mediated by ZEB1 in Cancer Cells

ZEB1 has been shown to promote metastasis of colorectal cancer cells [35]. Upon ZEB1 knockdown, there was a strong reduction of lung metastasis after splenic injection of tumor cells in nude mice. 

Another role of ZEB1 in cancer cells may be the circumvention of replicative senescence. Loss of ZEB1 in mice led to a MET process accompanied by premature senescence mediated by p15INK4b and p21 in mouse embryonic fibroblasts [36]. These observations have not been validated in carcinoma cells, however.

### 4.3. ZEB1 Expression in Human Tumor Tissue

With respect to its function in cancer cells, an important question is where ZEB1 is expressed in human tumor tissue samples. For carcinomas of colorectal, breast, lung, renal, endometrial and prostate origin, ZEB1 expression has been shown to be restricted to dedifferentiated tumor cells. These can be found at the invasive front of well-differentiated cancers or more widely distributed in the case of high-grade cancers [16,28,37,38,39,40,41]. Therefore ZEB1 expression usually correlates with EMT features, high tumor grade and poor prognosis.

The tumor-associated activated stroma seems to show a high ZEB1 expression in all carcinomas studied and can serve as a positive control.

## 5. ZEB1 in Pancreatic Cancer

### 5.1. ZEB1 Expression in Human Pancreatic Cancer Tissue

ZEB1 expression can be found in the epithelial cancer cells as well as the tumor-associated stroma. Similar to other cancers (see above), ZEB1 expression is usually restricted to undifferentiated cells, and accordingly an exclusive expression pattern of ZEB1 and E-Cadherin has been confirmed [42]. ZEB1 expression was more often found in a patient group with early recurrence than in the group with long-term remission after surgery [26].

### 5.2. Antagonism of ZEB1 and the miR-200 Family

The negative feedback loop between ZEB1 and members of the miR-200 family could also be demonstrated for pancreatic cancer cell lines [31]. A cell-line based microRNA screening in 21 pancreatic ductal carcinoma cell lines confirmed the existence of this regulatory loop in pancreatic cancer on a larger scale [43]. Furthermore, high miR-200c expression correlated with E-Cadherin expression and better survival after surgery for pancreatic cancer [44].

### 5.3. ZEB1 and Cancer Stem Cell Properties

When it became increasingly evident that EMT plays a major role in cancer invasion and metastasis, the Migrating Cancer Stem Cell hypothesis was formulated [45], which postulates the EMT process to be linked to an induction of cancer stem cell (CSC) properties. The first experimental evidence in favor of this hypothesis was presented by Mani and colleagues [46]. The authors demonstrated that EMT in Ras-transformed HMLER cells, induced by overexpression of Snail1 or Twist1, led to the acquisition of cancer stem cell traits like increased mammosphere formation, breast CSC surface marker expression and most importantly, increased tumorigenicity *in vivo*.

**Figure 2:**
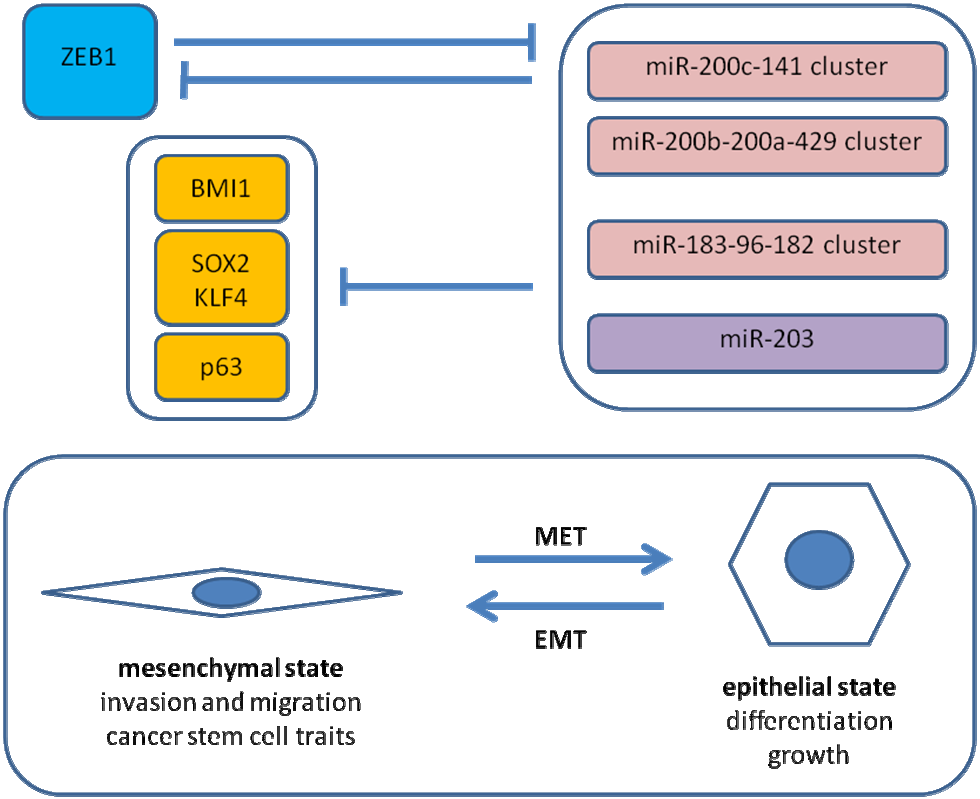
ZEB1-microRNA interactions in EMT. Shown is a simplified scheme of regulatory loops in EMT of cancer cells. A direct negative feedback loop between ZEB1 and the miR-200c-141 cluster stabilizes either the epithelial or the mesenchymal state. ZEB1 expression results in downregulation of the miR-200 family, miR-203 and miR-183. MiR-200c, miR-203 and miR-183 cooperatively inhibit BMI1 expression. SOX2 and KLF4 are putative targets of miR-200c. MiR-203 inhibits p63 expression.

Recently, we reported similar observations for EMT induced by ZEB1 in pancreatic cancer [26]. Knockdown of ZEB1 in two highly aggressive pancreatic cancer cell lines resulted in a reduction of tumorigenicity in limiting dilution assays as well as reduced tumor dissemination in an orthotopic nude mouse model. *In vitro*, tumor sphere formation and cancer stem cell surface marker expression were diminished. On the molecular level, a causal involvement of microRNA-repression by ZEB1 was demonstrated (shown in Figure 2). ZEB1 can inhibit expression of miR-200c, miR-203 and miR-183, which in turn were shown to target BMI1, a known factor for stem cell renewal. This regulatory pathway seems at least in part to be evolutionally conserved. Regarding other stem cell factors, putative conserved targets of miR-200c comprise KLF4 and SOX2, and p63 is a known target of miR-203. Therefore, it is proposed that ZEB1 expression results in EMT and micro-RNA repression leading to disinhibition of stem cell factors, hence stem cell traits. Inhibition of tumor sphere formation by the respective micro-RNAs could be demonstrated for long established and low-passage human pancreatic cancer cell lines as well as a mouse pancreatic cancer cell line.

The described regulatory network may also apply to other stem cells, as shown for mouse embryonic stem cells [26]. Intriguingly, downregulation of the two miR-200 family clusters and miR-183-96-182 cluster has also been found to be a common feature of mouse and human mammary epithelial stem cells and breast cancer stem cells. BMI1 is likely also regulated by miR-200c in these models. Overexpression of miR-200c inhibited mammary outgrowth from breast epithelial cells in mice, clonogenicity of mouse breast cancer cells, growth of embryonic carcinoma cells and tumorigenicity of human breast cancer stem cells [47]. It has been postulated that the micro-RNA-200 family acts as a universal suppressor of pluripotency [48]. However, this concept is challenged by the recent observation that a mesenchymal-epithelial transition, accompanied by expression of the miR-200 family and miR-205, is necessary for the generation of induced pluripotent cells (iPC) from fibroblasts [49,50].

### 5.4. ZEB1 and Oncogene Addiction

Singh and colleagues performed a comprehensive study of K-ras dependence in a panel of K-ras mutant lung and pancreatic carcinoma cell lines [51]. Epithelial differentiation correlated with K-ras dependence whereas cell lines with signs of EMT did not undergo apoptosis in response to K-ras knockdown. Accordingly, knockdown of ZEB1 in K-ras independent cell lines resulted in E-Cadherin re-expression and K-ras addiction. On the other hand, TGFß1-induced EMT could confer independence of K-ras. A “K-ras addiction signature” developed from these cell lines showed significant association with signatures derived from earlier studies of genes overexpressed in pancreatic cancer tissue samples. In summary, these observations suggest that the majority of human pancreatic cancer cells are addicted to K-ras, but K-ras dependency can be overridden by ZEB1-induced EMT. This is also in line with the observation that EMT causes resistance to EGFR-inhibitory drugs in pancreatic cancer [52,53].

### 5.5. ZEB1, EMT and Drug Resistance

Several studies have shown an association of ZEB1 expression with resistance to drugs used to treat pancreatic cancer [26,42,51,52,54,55]. This topic is discussed separately, although resistance to therapy also is a feature of CSC.

A comprehensive analysis of multidrug chemoresistance in pancreatic cancer was reported by Arumugam and colleagues [42]. Gene expression analysis of nine cell lines revealed a correlation of ZEB1 expression and loss of E-Cadherin with resistance against Gemcitabine, 5-FU and cis-Platin. Knockdown of ZEB1 led to an increased chemosensitivity, confirming a causal role of ZEB1-mediated EMT for multidrug resistance.

Wang *et al.* [55] analyzed pancreatic cancer cells that had been exposed to gemcitabine for several months *in vitro*. The emerging population of chemoresistant cells was found to have undergone EMT and displayed a high expression of mesenchymal markers and ZEB1, as well as increased migration and invasion [56]. The fraction of cells triple-positive for pancreatic CSC surface markers (CD44, CD24, ESA) was greatly increased, suggesting an enrichment of CSC by long-term exposure to chemotherapy [56]. The EMT features and ZEB1 expression could be partly reversed by transient knockdown of Notch2 and Jagged1, which also reduced cell migration and invasion. We made similar observations after gemcitabine-selection in another cell line and showed that miR-200c is lost in the gemcitabine-resistant cells. In addition, tumor sphere formation was greatly increased, confirming the link between CSC traits and EMT [26].

While the association of EMT and ZEB1 expression with drug resistance seems to be clear, the exact underlying molecular mechanisms have yet to be elucidated. For example, repression of miR-200c by ZEB1 results in disinhibition of TUBB3, which in turn is responsible for resistance to microtubule-targeting agents [57].

Several lines of evidence draw further attention to a role for ZEB1 and EMT in acquired drug resistance. A recent sophisticated analysis by Sharma *et al*. [58] implicated chromatin remodeling mediated by IGF1-signalling, HDAC and methyltransferase activity. IGF1-induced EMT has been shown to involve upregulation of ZEB1 [41]. The ZEB proteins assemble with HDAC1/2 and methyltransferases to form the CtBP core complex [15]. E-Cadherin [27] as well as the miR-200c-141 cluster [59] have been reported to be strongly regulated through DNA methylation.

## 6. Conclusions

ZEB1 and the microRNA-200 family have been identified as central players in regulation of EMT, invasion and metastasis of pancreatic and other cancers. EMT has been shown to be linked to the acquisition of CSC traits, as postulated by the concept of Migrating Cancer Stem Cells. An increasingly recognized feature of EMT and CSC is resistance to anticancer agents, and ZEB1 has been implicated in drug resistance of pancreatic cancer cells. Hopefully our increasing knowledge on the molecular basis of treatment resistance and metastasis will help to treat pancreatic cancer more efficiently in the future.
